# Eosinophil-derived neurotoxin: A biologically and analytically attractive asthma biomarker

**DOI:** 10.1371/journal.pone.0246627

**Published:** 2021-02-10

**Authors:** Bert Rutten, Simon Young, Magdalena Rhedin, Marita Olsson, Nisha Kurian, Farhat Syed, Augusta Beech, Mark Fidock, Paul Newbold, Dave Singh, Adam Platt, Glen Hughes

**Affiliations:** 1 Precision Medicine, Oncology R&D, AstraZeneca, Cambridge, United Kingdom; 2 COPD/IPF Bioscience, Research and Early Development, Respiratory & Immunology, Biopharmaceuticals R&D, AstraZeneca, Gothenburg, Sweden; 3 Early Respiratory & Immunology Statistics, Biopharmaceuticals R&D, AstraZeneca, Gothenburg, Sweden; 4 Medicines Evaluation Unit, University of Manchester, Manchester University NHS Foundation Hospital Trust, Manchester, United Kingdom; 5 Late Stage Respiratory and Immunology, Biopharmaceutical R&D, AstraZeneca, Gaithersburg, Maryland, United States of America; 6 Translational Science and Experimental Medicine, Research and Early Development, Respiratory & Immunology, Biopharmaceuticals R&D, AstraZeneca, Cambridge, United Kingdom; National and Kapodistrian University of Athens, GREECE

## Abstract

There is a growing body of evidence for the utility of eosinophil-derived neurotoxin (EDN) as a biomarker in asthma, including association with eosinophilic airway inflammation, assessment of disease severity and potential for predicting pathogenic risks, including exacerbations. However, to interpret any biomarker data with confidence, it is first important to understand the preanalytical factors and biological variation that may affect its reliable measurement and results interpretation. In this study we defined the healthy serum EDN reference range for men and women as 1.98 to 26.10 ng/mL, with no significant gender differences. Smoking did not impact the mean EDN levels and no circadian rhythm was identified for EDN, unlike blood eosinophils (EOS) where levels peaked at 00:00h. EDN expression in different cell types was investigated and shown to occur primarily in eosinophils, indicating they are likely to be the main cellular repository for EDN. We also confirm that the quantification of serum EDN is not influenced by the type of storage tube used, and it is stable at ambient temperature or when refrigerated for at least 7 days and for up to one year when frozen at -20°C or -80°C. In summary, EDN is a stable biomarker that may prove useful in precision medicine approaches by enabling the identification of a subpopulation of asthma patients with activated eosinophils and a more severe form of the disease.

## Introduction

In 2015, the global number of asthma sufferers was estimated to be 352 million people, an increase of ten percent over a decade [1]. Asthma is characterized by chronic airway inflammation resulting in a variety of respiratory symptoms including wheezing, shortness of breath, chest tightness and cough [2]. It is now accepted that asthma is a heterogeneous disease in nature, consisting of many different overlapping clinical phenotypes and molecular endotypes [3, 4]. This heterogeneity and complexity underpin the variability seen in therapeutic treatment responses [5, 6] requiring new precision medicine approaches to identify and treat these clinical subpopulations.

Eosinophilic inflammation is a common feature in many asthma patients, and is associated with a higher risk of a more severe disease [7], where elevated levels of eosinophils in the lungs and blood may be observed. Activated eosinophils release cytokines, lipid mediators, chemotactic polypeptides and cytotoxic proteins from their granules that neutralise pathogens as well as propagate a proinflammatory host response [8]. The cytotoxic proteins include major basic protein, eosinophil peroxidase, eosinophil cationic protein and eosinophil-derived neurotoxin. EDN is an RNAse with broad anti-viral and anti-bacterial activity that may also promote further leukocyte activation and is implicated in the pathophysiology of asthma [9, 10]. EDN can serve as a useful biomarker of eosinophil cell activation and degranulation in asthma patients. The measurement of eosinophil degranulation may be biologically more important than eosinophilia in asthma and therefore, it may not be the total number of eosinophils present that is important, but whether they are activated [11].

A number of studies have looked at the role of EDN and its use as a clinical biomarker for the diagnosis and monitoring of asthma. Serum EDN correlates with asthma disease severity [12] and with airway hyper-reactivity [13]. Elevated levels of EDN have been observed in asthma patients, with higher levels being measured during asthma exacerbations compared to those with stable asthma [14]. Furthermore, sustained exposure to high levels of EDN may have detrimental effects on the surrounding tissues with EDN being linked to airway remodelling in eosinophilic chronic rhinosinusitis by inducing production of matrix metalloproteinase 9 [15]. This suggests that prolonged airway exposure to elevated levels of EDN may be a risk factor for the progression of disease pathology. Measuring EDN levels could also be useful for monitoring therapeutic effect and asthma control. Levels of EDN have been shown to decrease after treatment with budesonide and benralizumab [16, 17].

EDN is potentially an analytically attractive biomarker that can be reliably quantified, unlike other eosinophil granule proteins, which are inherently sticky due their strong polycationic properties [14, 18]. Quantitative measurement of EDN can be achieved in a number of biological fluids including serum, plasma, urine, bronchoalveolar lavage fluid and sputum [12, 19, 20]. However, the concordance of EDN in these sample matrices is not clear. To interpret any biomarker data with confidence, it is first important to understand the preanalytical factors that can impact the biomarker stability and the natural biological variation that can occur. In this study we sought to determine the stability of EDN whole blood and serum samples, the healthy reference interval, the impact of smoking and atopy, the effect of circadian rhythm and the levels of EDN in key cell types.

## Materials and methods

### Subjects and serum sample collection

The EDN serum reference interval was established using 120 healthy volunteer samples from Bioreclamation, USA. For the temporal, smoking and atopy studies we recruited additional subjects, males or females aged between 18–75 years, from Medicines Evaluation Unit, Manchester University NHS Foundation Trust (MFT), UK. Written informed consent was obtained from all participants (research ethics committee REC reference 10/H1016/25 approved by NRES Committee North West, Preston, UK) and the study was conducted in accordance with the UK Human Tissue Act (2004) and the 1964 Declaration of Helsinki.

Healthy subjects had no history of hyper-eosinophilic disease (including hypereosinophilic syndrome, eosinophilic granulomatosis with polyangiitis, and eosinophilic esophagitis/gastritis) and any subject receiving oral corticosteroids or anti-eosinophilic therapies within the past 1 and 6 months, respectively, was excluded. Non-smokers had a pack year smoking history of <1 year and smokers had a history of ≥10 pack-years. Atopic subjects had a positive skin prick test, with a ≥3 mm wheal diameter to at least 1 of 3 common allergens (cat hair/dander, house dust mite and grass; ALK-Abello, Denmark).

Asthma patients had a physician diagnosis of asthma, requiring treatment with: (i) short-acting β2 adrenergic agonist alone or low dose inhaled corticosteroid (ICS) (<250 μg fluticasone dry powder formulation equivalents total daily dose) and a reliever medication or (ii) low dose ICS (>100 and <250 μg fluticasone dry powder formulation equivalents total daily dose) plus long-acting β2 adrenergic agonist ± theophylline, montelukast OR medium dose ICS (>250 and <500 μg fluticasone dry powder formulation equivalents total daily dose) and a reliever medication (mild and moderate asthma patients respectively), for at least 12 months prior to the study.

For serum preparation a venous whole blood sample was drawn into BD SST Vacutainers (12927696, ThermoFisher, UK) then allowed to clot at room temperature (22°C) for 30–60 minutes. Clotted blood was removed by centrifugation at 1500*g* for 10 minutes, at 4°C and serum stored in aliquots at -20°C and -80°C.

### EDN immunoassay

EDN in whole blood and serum was measured with a commercial EDN ELISA (30-EDNHU-E01, ALPCO, US) according to the supplier’s instructions. The microtitre plate, coated with capture anti-EDN monoclonal antibody, was washed 5 times with 250 μL of wash buffer in each well. After the final washing step, the residual buffer was removed by tapping the plate onto absorbent paper. Lyophilized calibration curve standards and controls were reconstituted in 500 μL ultrapure water. 100 μL of the calibration curve standards (0.25–16 ng/mL), controls (C1:[0.55 ng/mL] and C2:[2.63 ng/mL]) and samples (diluted 20-fold in assay buffer, giving an upper analytical measuring range of 320 ng/mL) were added in duplicate to the plate wells. The plate was covered tightly and incubated for 1 hour at room temperature (18–26°C) on a plate shaker. The contents of each well were then discarded and the plate washed as before. Residual buffer was removed and 100 μL of the detection anti-EDN peroxidase conjugated rabbit polyclonal antibody was added to each well and incubated for 1 hour. 100 μL substrate (tetramethylbenzidine) was added into each well and the plate was incubated for 15 minutes, at room temperature, in the dark. 100 μL acidic stop solution was added and the plate was read on a SpectraMax reader (Molecular Devices, US) at 450 nm against 620 nm as a reference. The EDN concentration of unknown samples were calculated from interpolation of the standard curve via a 4-parameter logistic fit using Softmax Pro GxP v5.0 software (Molecular Devices, US).

### Eosinophil blood cell count

Blood eosinophils were quantified as part of a complete blood cell count performed on a Sysmex XN-10 automated haematology analyser at MFT clinical laboratory (Manchester, UK). Cell counts were measured and reported to two decimal places.

### Tube equivalency study

Measurement of EDN recovery was conducted using five commercially available polypropylene tubes (8676, Corning; 5000–0020, Nalgene; 114842, Brand; 375418, Nunc; 126263, Greiner). 2 mL of 5 donor serum samples, covering the range 30–100 ng/mL EDN, were incubated in each tube type at room temperature for 2 hours.

### Stability of EDN in whole blood and serum

Human K_2_-EDTA anti-coagulated venous blood from 3 healthy subjects was sourced from a commercial supplier (Sera Labs, London, UK). Whole blood samples were left at room temperature on a tube roller until an aliquot was removed for EDN measurement at 0, 4, 24, 48 and 72 hours using the EDN immunoassay. Short term stability of serum EDN was assessed using seven serum samples at ambient temperature measured 1, 2, 3, 4 and 7 days after harvesting. Long term serum stability was assessed at baseline, 2 and 3 months, and then every 3 months for up to 1 year, at -20°C and -80°C. Freeze/thaw stability of human EDN in serum was determined at -20°C and -80°C after 1, 2 and 3 consecutive freeze/thaw cycles.

### Temporal changes in eosinophil and EDN levels

Quantification of matched circadian eosinophil and EDN variation in healthy volunteers (n = 10) and asthma patients (mild, n = 10; moderate, n = 10) was based on blood samples collected at time-points 16:00, 20:00, 00:00, 04:00, 08:00 and 12:00 hours.

### Cell preparation

#### Isolation of granulocytes and peripheral blood mononuclear cells

Donor blood (collected as part of ethical approval 033–10) was diluted with an equal volume of 2% Dextran (D8802, Sigma) in phosphate buffered saline pH7.4 (PBS; 10010, Gibco) and incubated until red blood cells (RBCs) had sedimented. Granulocytes and peripheral blood mononuclear cells (PBMCs) were isolated from the phase above the red blood cells by density gradient centrifugation using Lymphoprep^TM^ (07851, STEMCELL Technologies) as per the manufacturer’s instructions. In brief, the blood was layered onto an equal volume of Lymphoprep^TM^ and then centrifuged at 850*g* for 20 minutes at room temperature. The PBMC fraction was transferred to a new tube and washed once with buffer (PBS containing 2 mM EDTA and 2% heat inactivated FBS (15575, 10438, Gibco)) before isolation of monocytes. The granulocyte pellet was kept on ice and resuspended into 0.5 mL of cold PBS and 6.0 mL of water added to lyse the remaining RBCs. The lysis reaction was terminated after 15 seconds with stopping buffer (hypertonic PBS, 410 mM NaCl, pH7.4).

#### Sorting of eosinophils and neutrophils from granulocyte fraction

Granulocytes were washed once with PBS and stained with Live/Dead cell viability stain (L3496570, Invitrogen, ThermoFisher Scientific) for 15 minutes at room temperature. Cells were again washed with 4 volumes of cold PBS (10010, Gibco) and tubes spun at 350*g* for 10 minutes at 4⁰C and then diluted to 20 million cells/mL in PBS. 10–15 million cells were stained with anti-human CD16 AlexaFluor® 647 (561724, BD Bioscience) and anti-human CDw125 PE (555902, BD Bioscience) for one hour at 4⁰C. Cells were washed with PBS (as previously described), diluted to 15 million cells/mL in PBS and sorted on a SH800S Cell Sorter (SONY Biotechnology) with 100 μm microfluidic sorting chip. Eosinophil (CDw125^+^,CD16^-^) and neutrophil fractions (CDw125^-^,CD16^+^) were collected. The purity of sorted cell fractions was assessed with flow cytometry on a FACS Fortessa instrument (BD Bioscience; S1 Fig) before being spun down and lysed in buffer (0.056 M sodium acetate, 1 M NaCl, 1% Triton X-100, pH 4.5). Lysates were kept at -80⁰C until the determination of EDN content with the ALPCO immunoassay.

#### Enrichment of monocytes from PBMCs

Monocytes were enriched to high purity from PBMCs using magnetic cell sorting anti-CD14 coated beads as per manufacturer instructions (130–050201, Miltenyi Biotec, US). Purity was assessed with flow cytometry on a FACS Fortessa instrument (BD Bioscience). Isolated cells were spun down, lysed in buffer (0.056 M sodium acetate, 1 M NaCl, 1% Triton X-100, pH 4.5) and kept at -80⁰C until determination of EDN content with the ALPCO immunoassay. Purity was assessed with flow cytometry on a FACS Fortessa instrument (BD Bioscience). The data were analyzed using FlowJo software (FlowJo LLC; S1 Fig).

#### Hepatocyte preparation

Cryopreserved human hepatocytes (F00995-P, Celsis In vitro Technologies) were thawed and washed in CHRM medium (CM7000, Thermo Fisher) before being plated in INVITROGRO CP Medium (BioIVT). Cells were rested for 4 hours and then lysed in buffer (0.056 M sodium acetate, 1 M NaCl, 1% Triton X-100, pH 4.5). Lysates were kept at -80⁰C until analysis of EDN content with the ALPCO immunoassay.

#### Alveolar macrophage preparation

Alveolar macrophages were isolated from lung tissue obtained distal to tumour from patients undergoing lung resection, under informed consent and with ethical approval number 1026–15. Cells were flushed out from the tissue with PBS, collected by centrifugation and resuspended in Xvivo 10 medium (BE04-380Q, Lonza). Then cells were plated and allowed to attach for 1 hour before being washed to remove erythrocytes and incubated overnight at 37°C and 5% CO_2._ The next day cells were lysed in buffer (0.056 M sodium acetate, 1 M NaCl, 1% Triton X-100, pH 4.5) and kept at -80⁰C until analysis of EDN content with the ALPCO immunoassay.

### Statistical methods

Statistical evaluations (tests, confidence intervals, correlations) were based on log transformed EDN measurements, or log transformed eosinophil counts, due to the log-normal characteristic of this type of data. Presented group means and confidence intervals are back-transformed to linear scale. The reference interval was determined according to the Clinical Laboratory Standards Institutes guidance [21]. The reference interval is set at 95% of the upper and lower values measured from a population, meaning that the lower and upper reference limits are estimated as the 2.5^th^ and 97.5^th^ percentile. Temporal changes in EDN and eosinophils were analysed using a mixed model for repeated measurements, with log of EDN and log of eosinophils as response variables, timepoint and cohort (asthma, healthy) as factors with interaction, and subject as a random effect to account for within-subject correlation.

## Results

### Study subjects

80 healthy volunteers were enrolled into the smoker and atopic study. The non-smoker non-atopic subjects were 20–70 years (mean 46 years), 8 males and 12 females, 19 white and 1 mixed race; non-smoker atopic subjects were 21–62 years (mean 39 years), 9 males and 11 females, 17 white, 1 black and 2 mixed race; smoker non-atopic subjects were 41–70 years (mean 57 years), 6 males and 14 females, 19 white and 1 mixed race; smoker atopic subjects were 33–69 years (mean 52 years), 14 males and 6 females, 18 white, 1 black and 1 mixed race.

Healthy volunteers enrolled in the circadian study were aged between 24–67 years, 9 males and 1 female, all subjects were white and atopy negative; asthmatic subjects (50% mild and 50% moderate asthma) were aged 25–64 years and consisted of 13 males and 7 females, 16 white, 2 black and 2 mixed race, and 18 atopy positive. All asthma subjects had no exacerbations for the 12 months prior to or during the study. The mean Asthma Control Questionnaire (ACQ6) score was 0.62.

### Assessment of preanalytical variables

**Fig 1:**
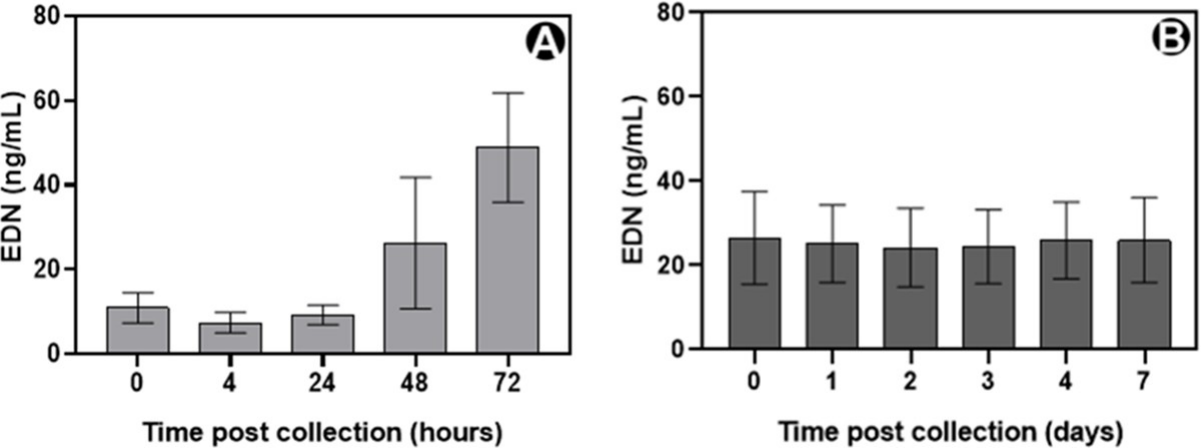
Stability of eosinophil-derived neurotoxin at ambient temperature. (A) venous whole blood from healthy subjects (n = 3) and (B) serum from healthy subjects (n = 7). Group means are shown with standard deviations.

We initially sought to confirm previous reports that EDN is not a sticky protein by testing the recovery of EDN protein from serum after incubation in different polypropylene storage tubes (Table 1). The coefficient of variation for each sample was 1.5 to 7.6%, indicating a low relative variability across the five tubes types. Next, we assessed the stability of EDN levels in both whole blood and serum from healthy volunteers. EDN levels did not change in whole blood for 24 hours, after which timepoint the concentration rose from 9.2 ng/mL (SD ± 2.3) to 48.9 ng/mL (SD ± 13.0), by 72 hours (Fig 1A), a relative increase by a factor of 5.3 (*P*<0.001). Conversely, ambient temperature serum EDN levels showed no significant changes from a mean value of 26.5 ng/mL (SD ± 11.0) at baseline to 26.0 ng/mL (SD ± 10.1) at day 7 (a relative mean reduction of 0.8%, 95%CI: -3.6%, 5.4%, Fig 1B). Refrigerated serum was equally stable for up to 7 days (Table 2).

**Table 1:** Tube equivalency study. 5 serum samples containing EDN were incubated with 5 different polypropylene tubes for 2hr at ambient temperature. ND: not determined.

Tube Type	EDN (ng/mL)				
	Subject 1	Subject 2	Subject 3	Subject 4	Subject 5
Corning	38.4	48.7	33.3	58.3	105.2
Nalgene	38.4	51.7	34.5	50.1	105.1
Brand	35.9	50.1	35.1	57.8	101.6
Nunc	39.3	ND	35.6	60.0	98.6
Greiner	38.4	ND	36.6	52.5	97.7
Mean (CV)	38.2 (3.5%)	50.2 (1.5%)	35 (3.5%)	55.7 (7.6%)	101.6 (3.5%)

**Table 2:** Stability of serum eosinophil-derived neurotoxin. Samples from 7 healthy volunteers were tested every day for short term stability and freeze/thaw, and every three months for long term stability. During the testing period the mean sample values should not exceed ± 15% the baseline value taken when the serum was prepared.

Stability Test	Testing period	Mean change from baseline at end of testing period
**Short term at 2–8**°**C**	7 day	-5.5%
**Long term at -20**°**C**	12 months	-7.6%
**Long term at -80**°**C**	12 months	-4.9%
**Freeze/thaw at -20°C**	3 cycles	-3.3%
**Freeze/thaw at -80°C**	3 cycles	-4.7%

To further asses the stability characteristics of serum EDN and to replicate real world use, samples were frozen and subjected to three repeated freeze/thaw cycles. Samples are considered to be stable if they stay within ± 15% of their baseline value, measured at the time of serum preparation and prior to freezing. Using this criterion, samples were stable for storage up to 12 months at either -20°C or -80°C (Table 2). However, the use of the ultralow temperature minimised sample degradation to 4.9% at -80°C, compared to 7.6% at -20°C. Serum samples were also stable for three freeze-thaw cycles, the maximum number we tested.

### Serum EDN reference interval

We measured serum EDN in 60 male and 60 female samples; the reference interval for men was 2.10 to 26.10 ng/mL and 1.98 to 25.71 ng/mL for women. The reference interval for men and women combined was 1.98 to 26.10 ng/mL.

### Effect of atopy and smoking on eosinophil and EDN levels in healthy subjects

**Fig 2:**
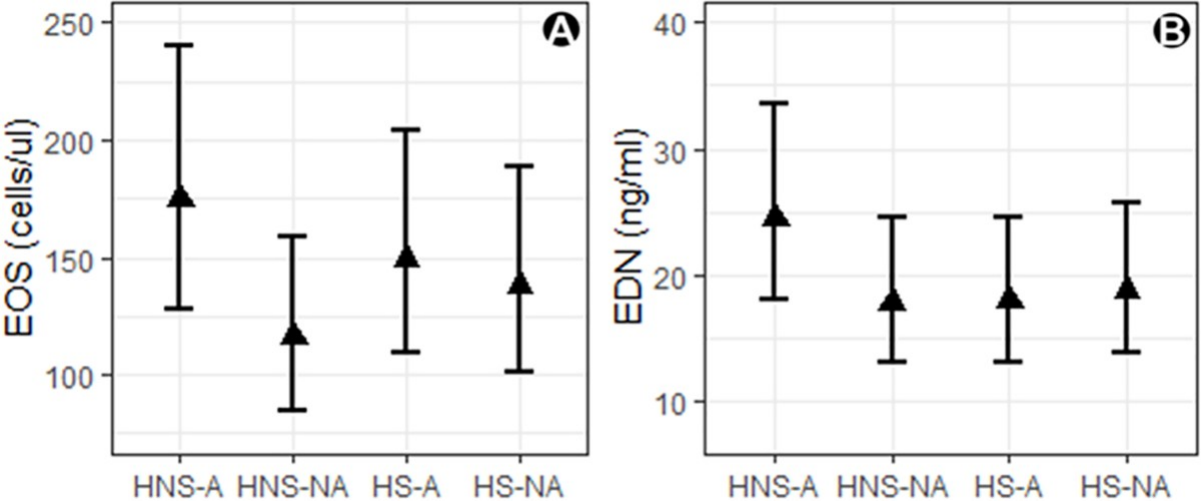
The effect of atopy and smoking on eosinophil and eosinophil-derived neurotoxin levels in healthy subjects. A) blood eosinophils (B) serum EDN where HNS-A, healthy non-smoker atopic; HNS-NA, healthy non-smoker non-atopic; HS-A, healthy smoker atopic; HS-NA, healthy smoker non-atopic. Group means are shown with 95% CI and each cohort had 20 subjects.

Of the four combinations of smoking and atopic status tested (Fig 2), the mean eosinophil and EDN levels were comparable for all, except for non-smoker atopic subjects who were higher and where there was a tendency for a significant mean difference in eosinophils (*P* = 0.07). A strong Pearson correlation coefficient (*r*) was observed between eosinophil and EDN levels (calculated on log-transformed data) in healthy subjects who were not smokers, whether without or with atopy; *r* = 0.85, 95% CI: 0.65,0.94 (*P*<0.0001) and 0.82, 95% CI: 0.60,0.93 (*P<*0.0001), respectively. Conversely, there was a weak to moderate correlation seen with smokers without or with atopy, *r* = 0.46, 95% CI: 0.03,0.75 (*P* = 0.03) and 0.35, 95% CI: 0.11,0.68 (*P* = 0.13), respectively (S2 Fig).

### Temporal changes in eosinophil and EDN levels in mild/moderate asthma patients

**Fig 3:**
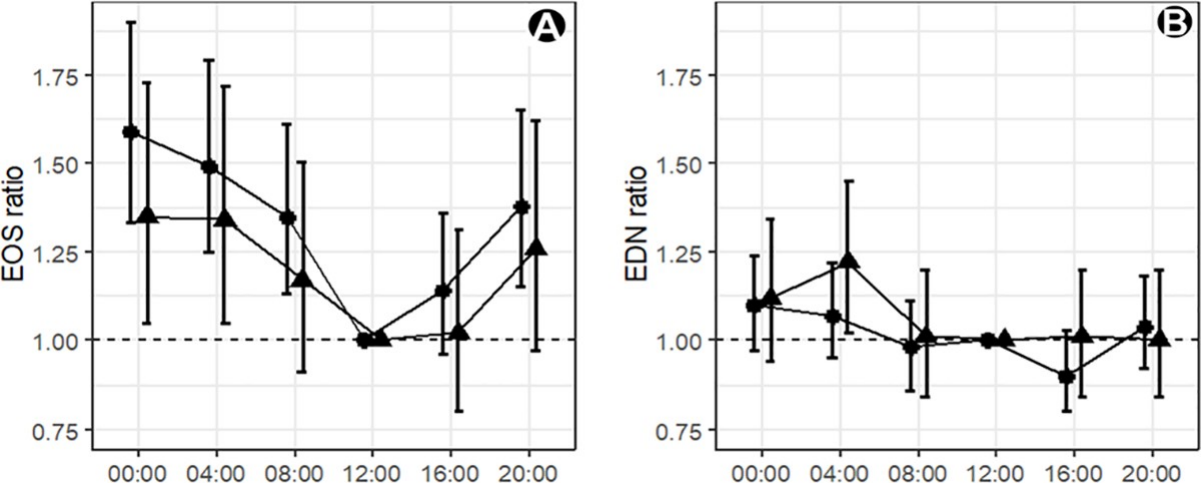
Temporal variation in eosinophil and eosinophil-derived neurotoxin levels. An EOS or EDN ratio of 1.00 indicates there is no difference in mean between that timepoint and the mean level at 12:00h for (A) blood eosinophils (B) serum EDN for (▲) healthy (n = 10) and (●) mild/moderate asthma subjects (n = 20). Group means are shown with 95% CI.

To assess possible time of day differences in blood eosinophils and serum EDN levels we sampled blood from subjects every four hours over a 24 hour period. For mild/moderate asthma patients, blood eosinophil levels showed a circadian rhythm, with a significant increase during the night and early morning (Fig 3A). The fluctuation peaked at 00:00h with a mean EOS cell count of 281 cells/μL and a relative ratio to the mean EOS at 12:00h of 1.59, 95% CI: 1.33,1.90 (*P*<0.001). Healthy subjects also displayed a time of day difference in EOS but with a smaller relative ratio at 00:00h of 1.35, 95% CI: 1.05,1.73 (*P* = 0.018). There was no clearly discernible EDN circadian rhythm in either asthma or healthy subjects, except for an increased mean in the asthma cohort at 04:00h relative to 12:00h in healthy subjects (Fig 3B) of 1.22, 95% CI: 1.02,1.45 (*P =* 0.027).

### Comparison of the cellular expression levels of EDN

**Fig 4:**
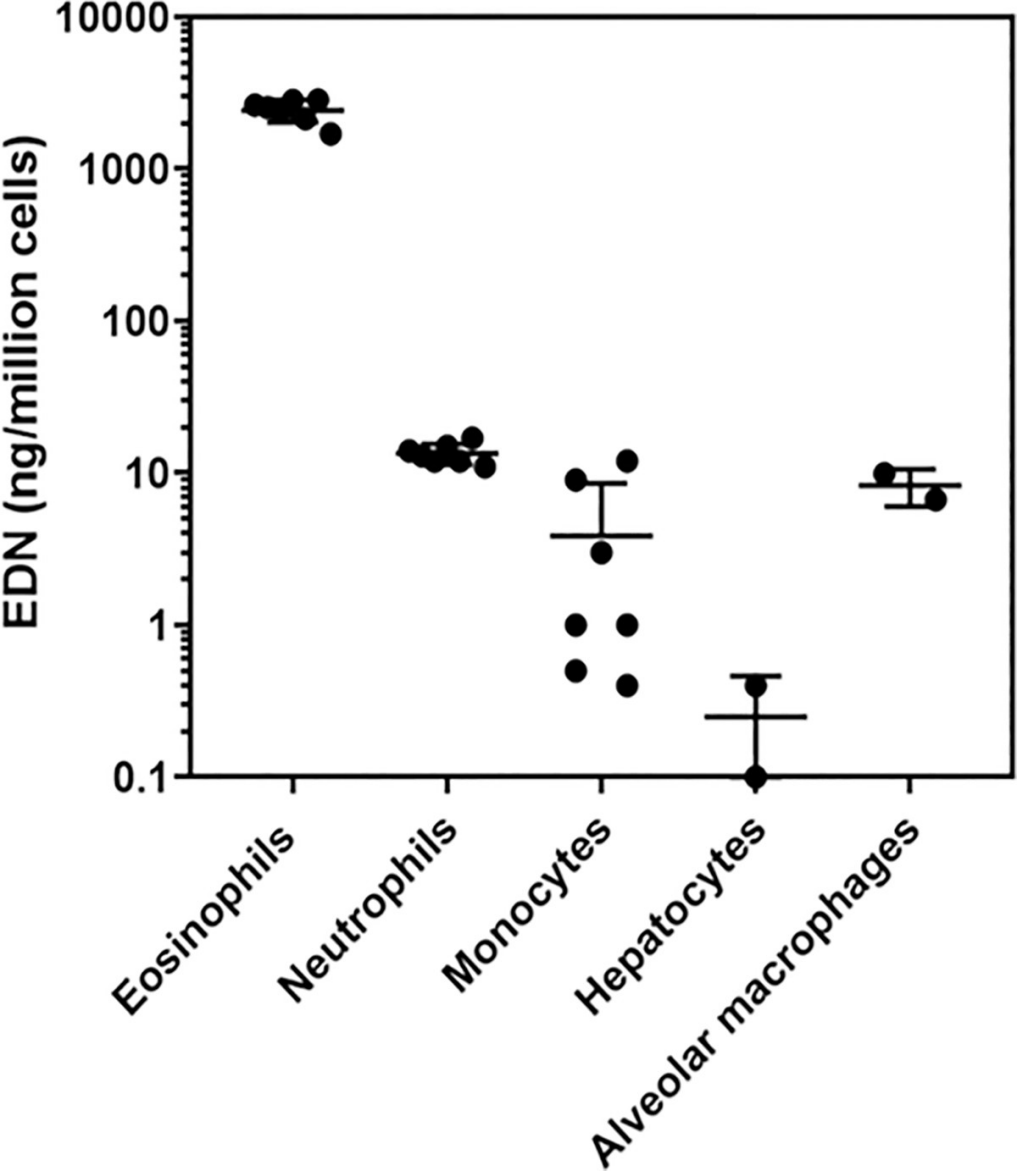
Scatter dot plot showing the comparison of eosinophil-derived neurotoxin levels in donor derived cells: Eosinophils, neutrophils, monocytes (n = 7) and hepatocytes and alveolar macrophages (n = 2). Group means are shown with standard deviations.

To identify potential cellular sources of EDN, mRNA levels in different cell types were first evaluated *in-silico* using transcriptomic datasets (RNA seq profiling databases at proteinatlas.org, gtexportal.org and blue-print-epigenome.eu). Based on these data and published information we chose cell types, in addition to eosinophils, that could be a possible EDN source contributing to serum levels. EDN protein measured in isolated cells as means ± SD are: eosinophils 2437.1 ± 407.9 ng/10^6^ cells; neutrophils 13.4 ± 2.1 ng/10^6^ cells; monocytes 3.8 ± 4.7 ng/10^6^ cells; hepatocytes 0.3 ± 0.2 ng/10^6^ cells; alveolar macrophages 8.3. ± 2.3 ng/10^6^ cells (Fig 4).

## Discussion

This study shows that serum EDN displays many characteristics of an ideal biomarker as it is easy to obtain and store, is readily quantifiable and shows stability over 1 year when samples are frozen. We defined the normal range, demonstrating low levels in the normal population. There was some evidence of higher levels in asthma patients (although not reaching statistical significance), while a strong correlation with eosinophil counts in non-smokers was demonstrated. Furthermore, the diurnal variation observed for blood eosinophil counts in asthma patients was less marked for serum EDN measurements.

As part of our body of evidence, we first considered the possibility of non-specific binding of EDN to different polypropylene storage tubes, a well-known phenomenon for protein and peptide biomarkers and one that can distort data comparison [22]. We observed that EDN recovery was consistent across the tube types assessed at all of the serum EDN concentrations chosen. Insignificant tube binding is also supported by the small percentage shifts observed in the short and long term stability studies relative to freshly prepared serum at baseline. These data indicate that EDN is not a sticky protein [14]. Tube binding and equivalency studies are rarely performed by researchers, yet they can have a fundamental impact on comparing data sets [23].

Next, we considered the stability of EDN in whole blood and serum. In short-term stability studies, serum samples were stable at ambient temperature and when refrigerated for at least seven days. This was the maximum timepoint we measured as this seemed a practicable duration for conducting laboratory experiments. However, it is likely that short term stability extends beyond this period. At ambient temperature, whole blood EDN levels were stable for up to 24 hours, after which the levels increased approximately fivefold. This increase may be caused by granulocyte degranulation or cytolysis and concomitant release of intracellular EDN. The whole blood stability profile is likely to be important when considering the time to transport and process venous blood into serum or plasma during clinical investigations, although cooling the sample may aid further control of sample degradation. For long-term storage, we found serum EDN was stable for at least a year when frozen and also through repeated freeze/thaw cycles. Further serum stability timepoints are ongoing to up to 2 years. These stability data should always be taken into account when analysing historical samples and special care needs to be taken to ensure they are measured within the known sample stability time frame.

To better understand where elevated and potentially pathophysiological levels of EDN may start we determined the reference interval for serum EDN in healthy individuals. Using the ALPCO immunoassay there were no significant gender differences in EDN levels measured, for men it was 2.10 to 26.10 ng/mL and 1.98 to 25.71 ng/mL for women. As far as we can ascertain this is the first published healthy reference gender data for EDN. However, we also recommend that each laboratory should establish their own matrix specific reference interval, especially when using alternative immunoassays, due to assay commutability [21, 12]. A future study comparing the performance of the different commercial assays would aid the comparison of data between different studies. It is noteworthy that all subjects had quantifiable EDN, suggesting a constitutively low level may always be present, perhaps as a first line of defence against viral infection [9]. The low number of non-white subjects in this study limits any conclusions on EDN differences between races. We further explored potential confounders to the reference interval range including atopy and smoking. The mean EOS or EDN levels of subjects who smoked, with or without atopy, were not dissimilar to healthy subjects. However, higher EDN and EOS was associated with subjects that had atopy, although these values were still within normal range. This is partly supported by evidence from Kwon et al. [24] who looked at the factors affecting blood EOS in a non-asthma population and found subjects with atopy, allergic rhinitis and smoking were associated with higher EOS, but values were at the upper limit of the normal range. We also noted that smoking worsened the intra-subject correlation between EOS and EDN levels.

We identified a circadian rhythm in eosinophil levels in subjects with asthma, with a minimum at midday and a maximum at midnight. These observations are broadly in line with those of Durrington et al. [25] who observed that sputum and blood eosinophil levels were at their highest at 04:00h, and that these elevations also correlate with timings for nocturnal asthma [26]. We did not see a circadian rhythm for EDN but there was an increase during the night and early morning. The lack of a detectable circadian rhythm for EDN compared to EOS may well reflect their distinct kinetics, with a longer half-life for the protein in blood which is estimated at 15 days [17] compared to 8–18 hours for eosinophils [27]. However, Panzer et al. [20] reported that there is an early morning (7am) increase in EDN, compared to late afternoon (4pm), in asthma subjects. Whilst both studies enrolled mild asthma patients, the Panzer study had asthma subjects who had significantly higher levels of EDN levels at the two timepoints tested. This may suggest that these subjects have primed and activated eosinophils that have a greater propensity to degranulate and the differences require further clinical investigation.

Lastly, we considered the role that different cellular expression patterns could have on the utility of EDN as a biomarker of activated eosinophils. *In-silico* transcript profiling identified neutrophils, monocytes, hepatocytes and macrophages as potential sources of EDN. The EDN levels we measured from purified blood eosinophils were consistent with those previously reported [28]. Eosinophil EDN was approximately two-hundred fold higher than the other cell types, indicating that they are likely to be the major reservoir and likely contributor to EDN levels in the blood.

In conclusion, our results suggest that EDN is an analytically attractive biomarker that can be reliably quantified and is robust enough to store long term. Additionally, the biomarker does not suffer from significant temporal biological changes that could affect the determination of possible thresholds for the diagnosis of eosinophilic driven diseases, estimation of disease severity or the determination of drug responses. Further studies using EDN as a biomarker in eosinophil associated diseases including asthma are warranted.

## Supporting information

S1 Fig Flow cytometry gating strategy and purity of cell preparations. Samples of sorted eosinophils, neutrophils monocytes were stained with antibodies for cell surface markers and analysed by flow cytometry on a Fortessa FACS instrument. Single cells in the cell gate of the FSC-H vs SSC-H plot were gated for specific markers: CDw125^+^/CD16^-^ eosinophils, 99.5% pure; CDw125-/CD16+ neutrophils, 99.8% pure; CD14+ monocytes, 97.0% pure.
(TIF)

S2 Fig Effect of atopy and smoking on the correlation of eosinophil cell and eosinophil-derived neurotoxin levels in healthy subjects. A) non-smoker non-atopic: *r* = 0.85, *P*<0.0001 B) non-smoker atopic: *r* = 0.82, *P*<0.0001 C) smoker non-atopic: *r* = 0.46, *P* = 0.03 D) smoker atopic: *r* = 0.35, *P* = 0.13. Correlations are based on log-transformed data.
(TIF)
