# Supplementary figures and images for: Eosinophil-derived neurotoxin: A biologically and analytically attractive asthma biomarker
Source: PLoS One. 2021 Feb 10;16(2):e0246627. doi: 10.1371/journal.pone.0246627 (PMC7875349; doi:10.1371/journal.pone.0246627)

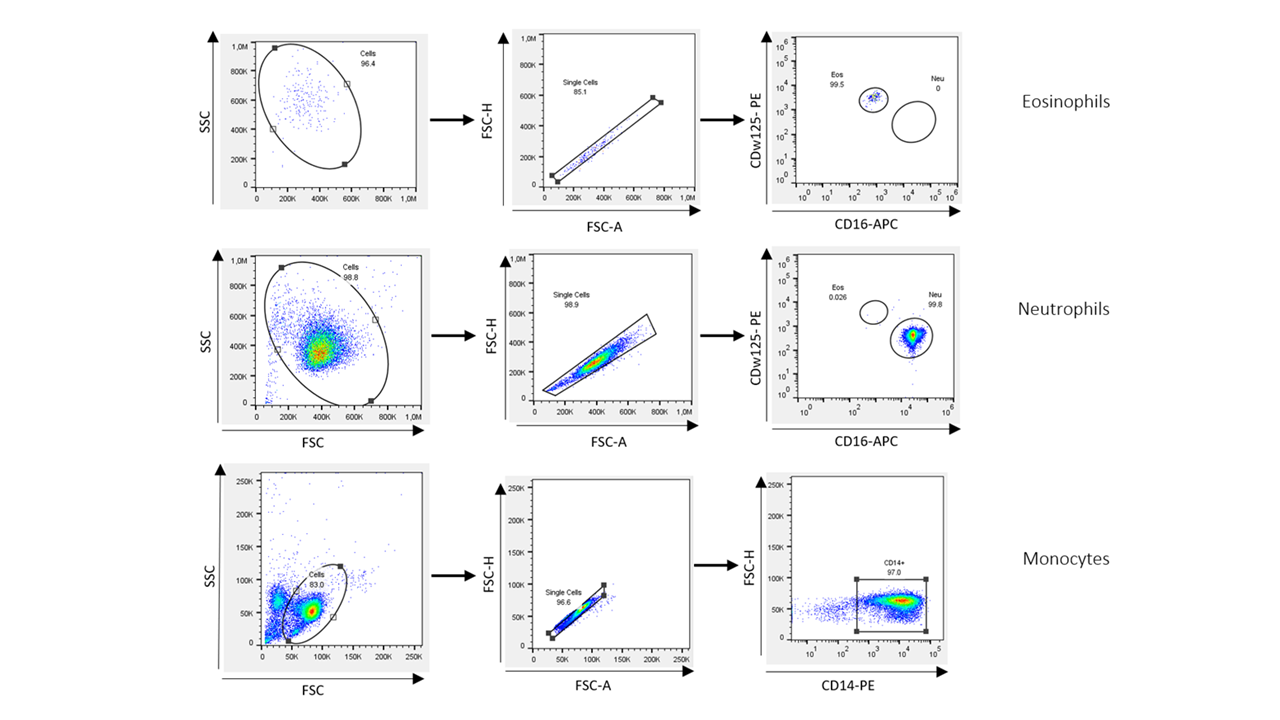

Supplement: S1 Fig — Samples of sorted eosinophils, neutrophils monocytes were stained with antibodies for cell surface markers and analysed by flow cytometry on a Fortessa FACS instrument. Single cells in the cell gate of the FSC-H vs SSC-H plot were gated for specific markers: CDw125+/CD16- eosinophils, 99.5% pure; CDw125-/CD16+ neutrophils, 99.8% pure; CD14+ monocytes, 97.0% pure. (TIF) [file pone.0246627.s001.tif]

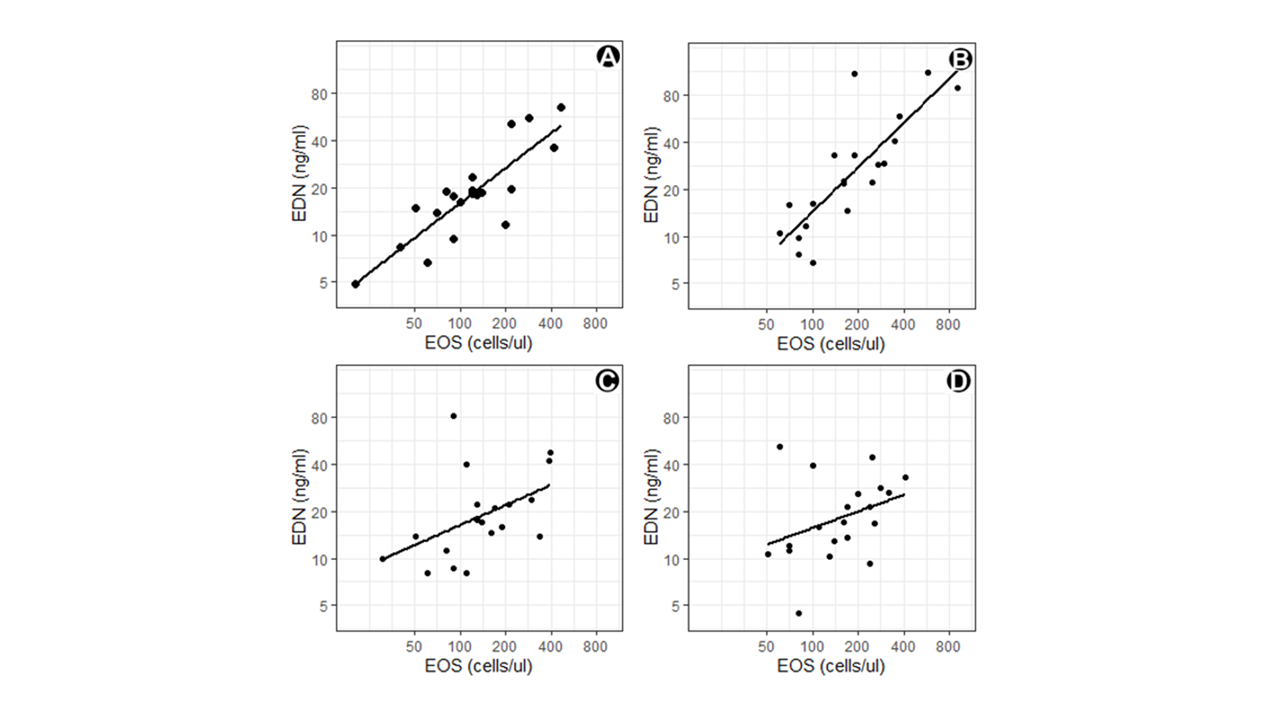

Supplement: S2 Fig — A) non-smoker non-atopic: r = 0.85, P<0.0001 B) non-smoker atopic: r = 0.82, P<0.0001 C) smoker non-atopic: r = 0.46, P = 0.03 D) smoker atopic: r = 0.35, P = 0.13. Correlations are based on log-transformed data. (TIF) [file pone.0246627.s002.tif]
